# *Isatis indigotica*: from (ethno) botany, biochemistry to synthetic biology

**DOI:** 10.1186/s43897-021-00021-w

**Published:** 2021-12-14

**Authors:** Jingxian Feng, Doudou Huang, Yingbo Yang, Junfeng Chen, Shi Qiu, Zongyou Lv, Xueqi Ma, Yuanyu Li, Rongrong Li, Ying Xiao, Wansheng Chen

**Affiliations:** 1 Research and Development Center of Chinese Medicine Resources and Biotechnology, The Ministry of Education (MOE) Key Laboratory for Standardization of Chinese Medicines, Institute of Chinese Materia Medica, Shanghai University of Traditional Chinese Medicine, Shanghai, 201203 China; 2 Jiangsu Kanion Pharmaceutical Co., Ltd, Jiangsu, 222001 Lianyungang China; 3 Medical Guarantee Center, Changzheng Hospital, Naval Medical University, Shanghai, 200003 China

**Keywords:** *Isatis indigotica*, Lignans, Indole alkaloids, Metabolic engineering, Synthetic biology

## Abstract

*Isatis indigotica* Fort. (Chinese woad) is a species with an ancient and well-documented history as an indigo dye and medicinal plant. It is often confused with *Isatis tinctoria* L. (European woad), a medicinal plant in Europe. Here, the differences between *I. indigotica* and *I. tinctoria* are systematically described. The usage development history, clinical applications and pharmacological activities, and chemical components of *I. indigotica* are also summarized. Lignans, indole alkaloids, and their corresponding derivatives have been identified as the major active ingredients of *I. indigotica* and are associated with anti-viral, anti-inflammatory, anti-cancer, and other health-promoting activities. Notable progress has been made in understanding the biosynthetic pathway and regulation mechanism of lignans and indole alkaloids in *I. indigotica*, the results from which should facilitate the process of targeted metabolic engineering or synthetic biology. Moreover, multiple biotechnology methods such as polyploid breeding and genetic engineering have been used with *I. indigotica* to result in**,** for example, greater yields, higher levels of bioactive component accumulation, and enhanced stress tolerance to salt, drought, and insects. Some issues require additional analyses, and suggestions for future research on *I. indigotica* are also discussed.

## Introduction

*Isatis indigotica* Fort. is a biennial herbaceous plant belonging to the Cruciferae (Brassicaceae) family and has a long history as one of the most prevalent Chinese medicinal herbs and dyes. To this day it continues to be used in medical treatments, the pharmaceutical industry, and the handmade textile industry. The root and leaf of *I. indigotica* are used to prepare three different traditional Chinese medicines. The dry roots, known as Banlangen (ISATIDIS RADIX), have anti-inflammatory and anti-viral activities, and can be further processed into Banlangen Keli. Both of these preparations are widely used for the treatment of flu and eruptive epidemic diseases in China (Zhang et al., 2016c). The dry leaves, called Daqingye (ISATIDIS FOLIUM), have pharmacological activity similar to that of Banlangen and are widely used for the treatment of fever, epidemic parotitis, and pharyngitis. Fufang Daqingye Heji, a liquid that is consumed orally, is the main preparation of Daqingye (Zhu et al., 2004). Qingdai (INDIGO NATURALS) is a more processed product of the leaves. It is the dry foam of fermented leaves and has a dark blue color, and is applied to the skin and used to heal wounds, acne, erysipelas, and carbuncles (Chen, 2013a).

Interestingly, the original plant species used for these three kinds of medicines did not consist solely of *I. indigotica*. Over time, *I. indigotica* gradually became the main cultivated species, mainly because of its larger output of materials and its better pharmacological activities. Currently, it is the only remaining species among the plants originally used to produce Banlangen and Daqingye and is the main plant component of Qingdai in the Chinese Pharmacopoeia (2020).

*Isatis tinctoria* L. (European woad) is a species in the same genus as *I. indigotica* (Chinese woad). The use of *I. tinctoria* in Europe also has a centuries-old history. Celtic and Germanic people used *I. tinctoria* as a source of indigo for dyeing and as a prophylactic in Roman times. From the 12th to the seventeenth century, *I. tinctoria* was widely promoted in Germany, France, England, and Italy (Hamburger, 2002; Speranza et al., 2020). Not until 2011, however, was it finally recognized as a medicinal plant in Europe (European Pharmacopoeia, 2011). Because of the similar uses and appearances of these two plants there has, however, been a long-standing dispute about the classification of *I. indigotica* and *I. tinctoria*. Some researchers have considered *I. indigotica* to be a synonym of *I. tinctoria*, although some taxonomists have confirmed that these two species have many differences in their morphology, genetics, and physiology (Angelini et al., 2007; Danz et al., 2001; Sun and Pang, 2002, 2013).

According to modern pharmacological investigations, *I. indigotica* possesses various biological activities, including its extracted parts and compounds. The active ingredients can be divided into several major groups; lignans and indole alkaloids and their corresponding derivatives, flavones, glycosides, and polysaccharides. A better understanding of these compounds and their biosynthetic pathways will extend our knowledge of the chemical characteristics of *I. indigotica*.

This review aims to clarify the recorded history of how *I. indigotica* gradually became the main plant in its related herbal preparations, to show the taxonomist’s point of view with respect to distinguishing *I. indigotica* from *I. tinctoria*, and to summarize the advanced knowledge of the pharmacologically-active secondary metabolites of *I. indigotica*. The biosynthetic pathways and key regulatory genes involved in the two major active ingredients, lignans and indoles, as well as biotechnology methods to improve *I. indigotica*, are reviewed and discussed.

## Historical use of *I. indigotica*

China is rich in plant species. The historical record shows that several species have been used for blue (indigo) dyeing, while also showing detoxifying activities. Such plants were referred to as Lan (indigo plant) in ancient China. These include Liaolan (*Polygonum tinctorium*) (Han, 2005; Jia et al., 1996), Songlan (*I. indigotica*) (Anonymous, 2008), Malan (*Baphicacanthus cusia*) (Su et al., 1994), and Mulan (*Indigofera tinctoria*) (Chen, 2004; Wang et al., 2020). The current Chinese Pharmacopeia describes Daqingye as the dry leaves of *I. indigotica*; Banlangen as dry roots; and Qingdai as the dry powder, agglomerates, or granules obtained by processing the aerial parts of *I. indigotica*, *P. tinctorium*, and *B. cusia* (Chinese Pharmacopoeia, 2020). The recorded components of these three traditional Chinese medicines have, however, changed over time.

A record of the medicinal use of Lan first appeared in the “Shen Nong Ben Cao Jing” (“Shen Nong’s Herbal Classic”) in the Spring and Autumn Period (770–475 BCE) (Anonymous, 2008). It is stated therein that the seeds of Lan, i.e. Lanshi, have the effect of detoxification. Huang and Chen (1965) reported that the Lan referred to in that ancient text was *I. indigotica*. In addition, *I. indigotica* was cultivated in the Late Eastern Han dynasty (?-CE 220) (Huang and Chen, 1965).

By the Wei, Jin, and Northern and Southern dynasties (CE 220–589), the leaves of some indigo plants with their seeds were included as antidotes for poison or insect poison. For this use, the juice processed from young leaves was the better choice (Tao et al., 2013; Wang et al., 2020). It should be noted here that the leaves used were just the leaves of Lan and were not specifically named Daqingye.

Daqing (*Clerodendrum cyrtophyllum* Turcz.) is a species from the family Verbenaceae. Daqingye was first used as the term to refer to a preparation of its leaves in ancient China; this preparation had similar efficacy to that of Lan and was used to treat aphtha. In the Qing Dynasty (CE 1636–1912), Yang Shitai, a famous physician, wrote in his book that Lan or Qingdai could be used as a substitute for Daqing when the latter was insufficient (Yang et al., 2009). From then on, Daqing and Lan were used interchangeably for medical use. Because of its greater curative effects and the larger amount of plant material it produced, Lan gradually replaced Qaqing as the main plant component of this medicine, although the name Daqingye was not changed (Wang et al., 2020; Yang et al., 2009).

Reference to Banlangen first appeared in the officially published books of traditional Chinese medicine in the Song dynasty (CE 960–1279), in which it was described as the root of Lan and having the same efficacy as the leaf. Banlangen became a kind of commonly used medicine in the Yuan dynasty (CE 1271–1368). The first original plant used to produce Banlangen was Malan (*B. cusia*), a specialty plant in Fujian province in China, where it was referred to as Banlan in the Fujian dialect. Therefore, the processed product derived from its root is referred to as Banlangen. During the late Qing dynasty, however, the original plant species used to produce Banlangen was confused with various Lan species (Huang and Chen, 1965; Teng, 1996). “Zhong Hua Ben Cao” (“Chinese Materia Medica”) documented Songlan (*I. indigotica*) as the original plant of Banlangen, and at that time *I. indigotica* had become the main cultivated species. Currently, the Chinese Pharmacopoeia (2020) defines Nanbanlangen (southern Banlangen) as the dry root and rhizome of Malan (*B. cusia*)

Qingdai originally referred to the powder extracted from shells imported from Persia, which also has a detoxifying effect, albeit with very low yield. It was discovered that the dry foam in the indigo dye vats had an effect similar to that of the powdered shell, and thus the dry foam came to replace the shell powder and also assumed its name (Teng, 1996).

Over the period of several dynasties it was determined that *I. indigotica* was indeed the best cultivated species for these medicines (Huang and Chen, 1965; Teng, 1996; Wu and Wang, 1996). This is a manifestation of the expression “practice is the only criterion for testing truth.”

## Differences between *I. indigotica* and *I. tinctoria*

**Fig. 1:**
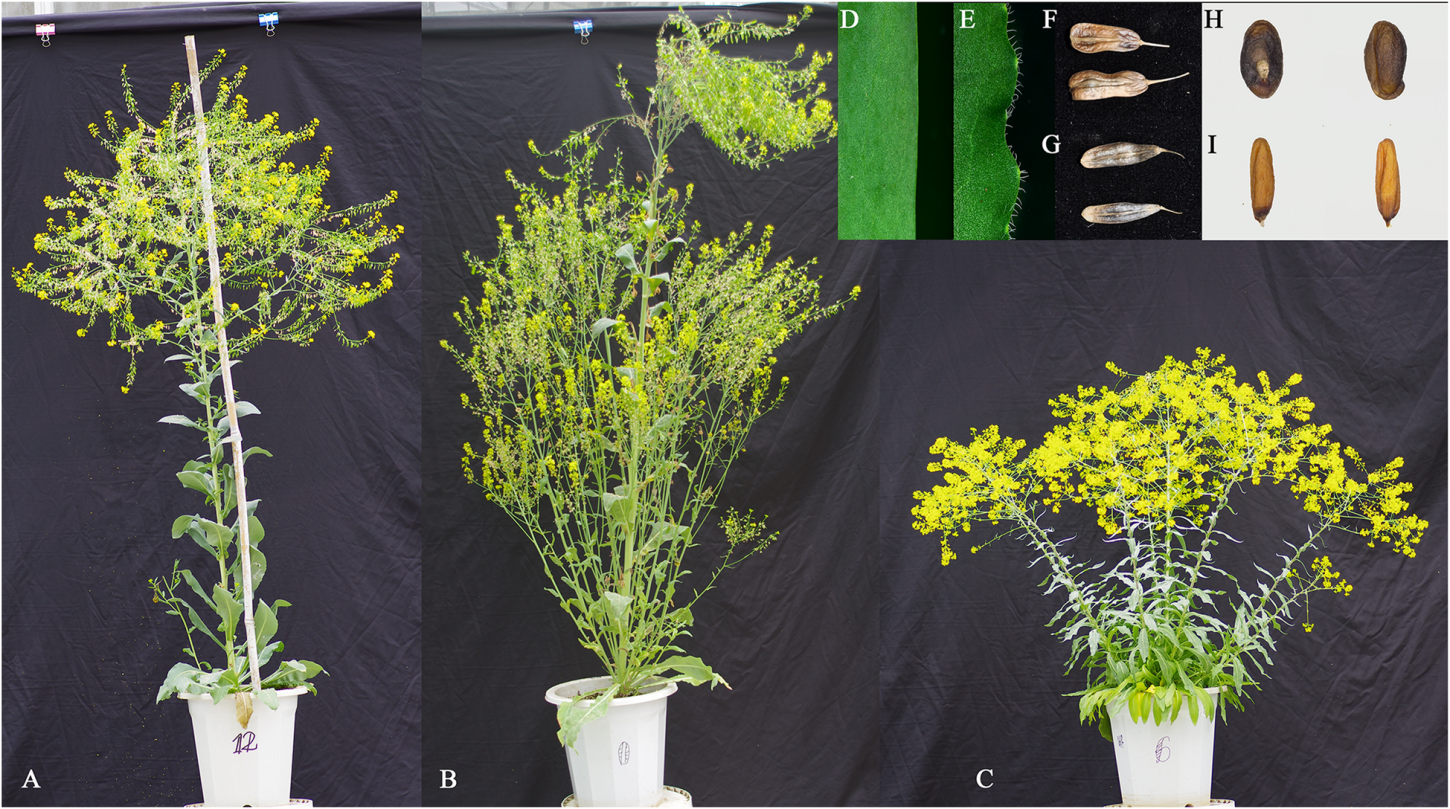
Morphological differences between *I. indigotica* and *I. tinctoria.* A; tetraploid *I. indigotica*; B; diploid *I. indigotica*; C; *I. tinctoria*; D, F, H; the leaf edge, fruits, and seeds of *I. indigotica*, respectively; E, G, I; the leaf edge, fruits, and seeds of *I. tinctoria*, respectively

Taxonomists conducted morphological, cytological, and palynological observations of *I. indigotica* and *I. tinctoria*, and analyzed their enzymes and metabolites. They concluded that *I. indigotica* differs from *I. tinctoria* and is native to China (Qiao, 1984; Wang and Zhou, 1982) (Fig. 1).

The seedlings and basal leaves of *I. indigotica* are light pinkish-gray, smooth, and hairless. The stem leaves are mostly oblong and also smooth and hairless. The petals are oblanceolate, with a claw-shaped base and truncated top. The fruits are oblong-ovate or oblong, wedge-shaped at the base, and blunt round or truncated at the top; their middle ribs are thin and inconspicuous, and some locules do not bulge. This species has seven pollen mother cells and 2n = 14 somatic cells (Qiao and Cui, 1982).

In contrast, the seedlings and basal leaves of *I. tinctoria* are blue, with unicellular, non-glandular hairs. The stem leaves are mostly long and lanceolate, with sparse unicellular, non-glandular hairs. The petals are obovate with an inconspicuous and claw-shaped base and round top. The fruits are mostly wedge-shaped, round, or acuminate at the base; their middle ribs are thin and inconspicuous, and the locules bulge conspicuously. This species has 14 pollen mother cells and 2n = 28 somatic cells (Qiao and Cui, 1982).

From the perspective of plant evolution, chromosomes usually change from low to high ploidy level, and less often in the other direction. If the two members of the genus *Isatis* are in fact the same species, the tetraploid form of *I. indigotica* should be similar to *I. tinctoria.* However, tetraploid *I. indigotica*, when induced by colchicine growth retardation, has wider and thicker leaves, and its flower organs, pollen grains, fruits, and stomata are larger than those of the diploid *I. indigotica*, with notable differences to *I. tinctoria.* Therefore, the merging of these two species cannot be supported.

As per their respective metabolites, Huang (2020) detected much higher levels of indole alkaloid derivatives, quinolines, and flavonoids (e.g. isovitexin, isovitexin-3″-O-glucoside-7-O-glucoside, isovitexin-3″-O-glucoside, isoscoparin-3″-O-sinapoylglucoside, and isoscoparin-3″-O-feruloylglucoside) in *I. indigotica* than that in *I. tinctoria*. In contrast, the organic acid derivatives accumulated to higher levels in *I. tinctoria* than *I. indigotica*. Although one species did not consistently outproduce the other with respect to these metabolites, the differences between these two species were constant.

## Clinical applications and pharmacological activities of *I. indigotica*

As one of the most popular herbal drugs in China, *I. indigotica* has been used most often for detoxification, against fever, and curing aphtha in the three forms of traditional Chinese medicine described above. In modern clinical applications, however, herbal extracts of *I. indigotica* and prescriptions or the isolated bioactive constituents have shown preventive and therapeutic effects against influenza and upper respiratory tract infection, inflammation, and allergies. For instance, a water-based extract of the roots can inhibit different subtypes of human and avian influenza viruses, including H1N1 and H3N2 (Haruyama and Nagata, 2013; Ho et al., 2014; Yang et al., 2012; Zhang et al., 2020). In combination with other herbal drugs, the leaves and roots of *I. indigotica* are used to treat icteric hepatitis, parotitis, condyloma, palmoplantar pustulosis, epidemic kerato-conjunctivitis, and viral myocarditis (Han, 2012; Li et al., 2007; Ma, 2003).

Numerous pharmacological studies have reported the activities of extracts or isolated compounds of *I. indigotica* both in vivo and in vitro. These preparations or compounds have anti-viral, anti-inflammatory, analgesic, anti-microbial, and even anti-tumor activities. The leaf extracts can inhibit infection and proliferation of influenza A, H7N9, encephalitis B, mumps viruses, HSV-II, Dengue virus II, and Cytomegalovirus, among others (Ma, 2014; Zhou et al., 2006); directly neutralize and degrade endotoxin in actinomycin D-sensitized mice (Shi and Zhang, 2006) and promote interleukin 2 (IL-2) secretion by spleen lymphocytes induced by concanavalin A to enhance immunity in normal mice (Hsuan et al., 2009; Liang et al., 2000). They also have anti-bacterial effects against *Staphylococcus aureus* and *Escherichia coli* (Lejars and Hajnsdorf, 1863) and promote bile excretion and relieve pain (Hong et al., 2010; Liu et al., 2009). The root extracts have similar activities, such as inhibiting the human H7N9 avian influenza virus by preventing the virus from invading the host cells; inhibiting HSV-1 by preventing its replication and proliferation in cells; inhibiting *E. coli*, *Staphylococcus epidermidis*, *Pneumococcus*, *Haemophilus influenzae*, and *Streptococcus*; and decreasing the levels of tumor necrosis factor-α (TNF-α) and IL-6 in peritoneal macrophages of mice (Hu et al., 2003; Wang et al., 2012). Ghosh et al. (2020) reported two potential SARS CoV-2 Mpro inhibitors, sinigrin and hesperetin, derived from the root of *I. indigotica*, using molecular docking. These two compounds interacted with the important catalytic residues of Mpro (His41 and Cys145), and might be considered for corona virus disease 2019 (COVID-19) treatment (Ghosh et al., 2020).

Various compounds isolated from *I. indigotica* have been reported as bioactive components, such as tryptanthrin, indirubin, lariciresinol, clemastanin B, epigotin, and polysaccharides. Tryptanthrin has been found by a few studies to have anti-inflammatory activity and inhibits breast cancer cell proliferation in vitro. It inhibited cyclooxygenase 2 (COX-2) and 5-lipoxygenase (5-LOX) in cell-based assays and inhibited *Trichophyton mentagrophytes*, which causes tinea pedis (Tang et al., 2004). Zeng et al. (2021) reported that tryptanthrin inhibited the proliferation, migration, and invasion of human breast adenocarcinoma Michigan Cancer Foundation-7 (MCF-7) cells, and regulated the level of related proteins in vitro. Tryptanthrin effectively inhibited tumor growth in 4 T1 murine breast cancer model; modulated expression levels of nitric oxide synthase 1 (NOS1), COX-2, and nuclear factor kappa-B (NF-κB) in mouse tumor tissues; and regulated some factors such as IL-2, IL-10, and TNF-α in mice (Zeng et al., 2021). Indirubin possesses anti-tumor activity as shown by its ability to inhibit the growth of transplanted tumors and alleviate chronic myeloid leukemia (Xu et al., 2010). Indirubin and its derivates can interrupt virus-induced kinase activation and NF-κB translocation (Ye et al., 2011), and inhibit cyclin-dependent kinase (CDK) and histone deacetylase (HDAC) in several cancer cell lines (Cao et al., 2021). Li (2015) determined that lariciresinol and its glycosylated products were useful for the treatment of influenza A1 virus. Yang et al. (2013) determined that a lariciresinol derivative, clemastanin B [7*S*,8*R*,8′*R*-(−)-lariciresinol-4,4′-bis-*O*-β-d-glucopyranoside], can block the translocation of nucleocapsid protein at an early stage of virus replication (Yang et al., 2013). Epigotin has a strong inhibitory effect on influenza A1 virus FM1 by reducing susceptibility to the virus via mitochondrial anti-viral signaling (Luo et al., 2019). Polysaccharides prevent the influenza virus from attaching to host cell surfaces (Yang et al., 2012; Zhao et al., 2008); inhibit Hepatitis B virus (HBV) in vitro by reducing extracellular and intracellular DNA levels of HBsAg, HBeAg, and HBV in HepG2.2.15 cells (Yong and Aisa, 2011; Zhao and Aisa, 2012); and promote specific immunity, non-specific immunity, humoral immunity, or cellular immunity effects (Grienke et al., 2019). More detailed clinical applications and pharmacological activities of *I. indigotica* were systematically reviewed by Chen et al. (2021a).

## Chemical components of *I. indigotica*

Phytochemical investigations of *I. indigotica* have led to the isolation of various natural compounds including alkaloids, lignans, flavonoids, phenolic acids, and polysaccharides. Ultra high performance liquid chromatography quadrupole/time of flight-MS/MS (UHPLC-Q/TOF-MS/MS) studies of *I. indigotica* identified 116 indole alkaloids and their derivatives, 20 lignans and their derivatives, 105 carbohydrates and carbohydrate conjugates, 45 quinolines, 28 tetrapyrroles, 50 flavonoids, 35 alkaloids and their derivatives, 104 organic acids and their derivatives, and 360 lipid and lipid-like molecules (Huang, 2020). More than 150 of these components have been assessed in phytochemical pharmacology studies. Indole alkaloids and lignans, along with their corresponding derivatives, are considered the two major bioactive ingredients of *I. indigotica*.

Alkaloids in *I. indigotica* fall into two categories; indole and quinoline alkaloids. Indigo and indirubin, the two main indole alkaloids, have long been used as blue and red dyes, respectively, in China (Lu et al., 2012). Historically, *I. indigotica* was prepared as a decoction with water; more recently, researchers have used extraction with ethanol and methanol, which resulted in the isolation of several polar indole alkaloids that contained more than one glycoside. Six new diglycosidic indole alkaloid derivatives, referred to as isatigotindolediosides A-F, were isolated from dried roots of *I. indigotica* (Meng et al., 2017a). Another interesting class of indole compounds was discovered from aqueous extraction of *I. indigotica* (Meng et al., 2017b), in which there is a free sulfonic acid group. The sulfonic acid group not only increases the water solubility of the compounds but also enhances the anti-viral activity of the plant. As *I. indigotica* is the source of many mono-indole-related compounds, these compounds with their free indole groups can be naturally polymerized into more-complex indole-related compounds. In 2012, a previously uncharacterized molecule with linkages between 2-(4-methoxy-1*H*-indol-3-yl) acetonitrile and 2-(1*H*-indol-3-yl) acetonitrile was isolated from *I. indigotica* root (Chen et al., 2012), which was an enriched indole alkaloid type. Since then, a series of compounds with indole dimers have been isolated and identified from *I. indigotica* (Liu et al., 2015). Another important alkaloid type is the quinoline alkaloids, which contain a benzopyridine ring. Quinoline compounds in *I. indigotica* are also diverse and exist both as monomers and as polymers after polymerization with other groups. For example, (+)-(*R*)-2-oxo-1,2,3,4-tetrahydroquinoline-4-carboxamide was found to contain a single quinoline group, whereas isatisindigoticanine B contains a quinoline and an indole group (Zhang et al., 2019a).

Lignan has long been regarded as an effective anti-viral component in *I. indigotica*. Zhong’s laboratory reported that the two most common lignans, lariciresinol-4-*O*-β-d-glucopyranoside and clemastanin B, significantly decrease the pathogenicity of the virus H1N1 (Li et al., 2015; Yang et al., 2013). In an effort to identify additional bioactive lignans, 17 lignans were identified in an ethanol extract of *I. indigotica* (Zhang et al., 2019b). Lignans have long been considered to be formed by the formation of a C6-C3 junction between two monomers, although several lignans, consist of three monomer molecules, including isatindigosesquilignans A and B. It seems that the third monomer was also added by C6-C3 junction on the base of the dimer. The isolation of lignans with different molecular structures will help clarify their synthesis mechanisms.

## Biosynthetic pathways and metabolic regulation of bioactive compounds in *I. indigotica*

Previous studies based on transcriptomes revealed candidate genes for the biosynthesis of types of active compounds in *I. indigotica*. Chen et al. (2013b) reported a database of 36,367 isogenes generated by performing transcriptome sequencing of the hairy roots of *I. indigotica*, which are induced by *Agrobacterium tumefaciens*; they identified 104 unigenes involved in the general pathways of active compounds. However, the number of identified genes was limited, the expression level of the genes showed only the phenotypes of the various lines, and genes from hairy roots may not display all the characteristics of *I. indigotica* plants. Kang et al. (2020) reported a chromosome-scale genome assembly of *I. indigotica* with a total size of 293.88 Mb and scaffold N50 = 36.16 Mb. They annotated 30,323 protein-coding genes with high confidence, including 59 genes involved in terpenoid and sterol biosynthesis, 66 genes involved in biosynthesis of lignans and flavonoids, and 32 genes involved in biosynthesis of indole alkaloids. They suggested that numerous genes involved in the biosynthesis of the two major types of active compounds showed increased copy numbers because of tandem duplication, which may drive the production of active compounds.

### Biosynthesis and regulation of lignans

**Fig. 2:**
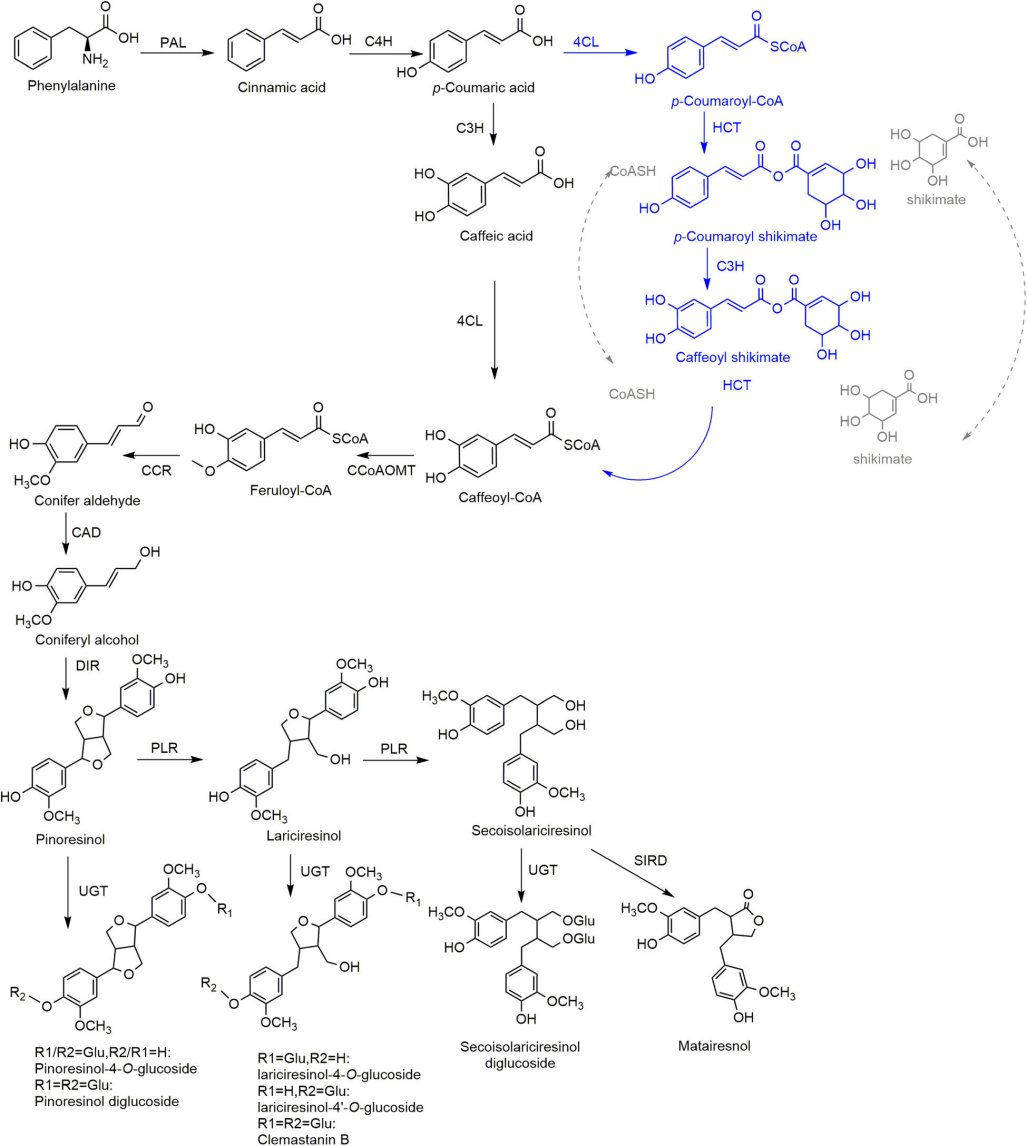
Biosynthetic pathway of lignans in *I. indigotica.* Lignin and lignans come from caffeoyl-CoA, which could be produced from p-coumaric acid through caffeic acid or from *p*-coumaroyl-CoA through the CoASH-shikimate cycle. Active lignans and their corresponding derivatives in *I. indigotica* initiate from the dimerization of coniferyl alcohol. Blue and gray routes indicate different biosynthetic pathway leading to caffeoyl-CoA. The figure is adapted from Xiao et al. (2015) and Chen et al. (2021b)

Lignans are a major class of phenylpropanoids, with two phenylpropane units connected through a β,β′-linkage, and are a family of secondary metabolites found widely in plants. The phenylpropanoid biosynthetic pathway in plants begins with phenylalanine. The 4-coumaroyl CoA is the bifurcation point of the metabolic flux. Flavonoids, isoflavonoids, phenylpropanoid esters, lignin, and lignans are all formed from this point. Lignin and lignans come from the same branch, in which *p*-coumaroyl CoA transfers into three kinds of monolignols, and then numbers of monolignols are converted to lignin. On a separate branch, the dimerization of coniferyl alcohol represents the initiation of lignan biosynthesis, and this branch competes with the lignin biosynthetic branch. One of the two furan nuclei in the pinoresinol structure is opened to generate lariciresinol, and then the other ring is opened to generate secoisolariciresinol. The two free hydroxyl groups reform into a five-membered ring to generate matairesinol. The four lignans in this flux can all be glycosylated with one or two glucose molecules on their free hydroxyl groups (Chen et al., 2021b) (Fig. 2, Table 1).

**Table 1:** Enzymes involved in lignan biosynthetic pathway of *I. indigotica*

Name	Enzyme	Gene ID	Substrate	Product	Reference
Phenylalanine ammonia-lyase	PAL1	–	Phenylalanine	*trans*-Cinnamic acid	Ma et al., 2016
	PAL2	–			
Cinnamate 4-hydroxylase	C4H	GU014562	Cinnamic acid	4-Coumaric acid	Hu et al., 2015
4-Coumarate CoA ligase	4CL	GQ872418	–	–	Di et al., 2012
	4CL2	KC430622	4-Coumaroyl acid; caffeic acid; ferulic acid; sinapic acid	4-Coumaroyl CoA;caffeoyl CoA et al.	Zhang et al., 2016a
	4CL3	KC430623	Caffeic acid; ferulic acid	Caffeoyl CoA; Feruloyl CoA	Zhang et al., 2016b
Coumaric acid 3-hydroxylase	C3H	JF826963	4-Coumaroyl CoA	Caffeoyl CoA	Chen et al., 2015
Caffeoyl CoA 3-O-methyltransferase	CCoAOMT	DQ115904	Caffeoyl CoA	Feruloyl CoA	Li et al., 2021
Cinnamoyl-CoA reductase	CCR	GQ872418	Cinnamoyl CoA esters	Cinnamaldehydes	Hu et al., 2011
Cinnamyl alcohol dehydrogenase	CAD	GU937874	Cinnamyl aldehydes	Cinnamyl alcohols	Hu, 2010
Hydroxycinnamoyl-CoA shikimate/ quinate hydroxycinnamoyl transferase	HCT	–	p-Coumaroyl CoA; caffeoyl shikimate	p-Coumaroyl shikimate; caffeoyl CoA	Dong et al., 2015
Dirigent protein	DIR1, DIR2	–	Coniferyl alcohol	Pinoresinol	Li et al., 2014; Chen, 2018
Pinoresinol/lariciresinol reductase	PLR1	JF264893	Pinoresinol; lariciresinol	Lariciresinol; secoisolariciresinol	Xiao et al., 2015; Xiao et al., 2021
UDP-glucuronosyltransferase	UGT71B5a	MW051594	Pinoresinol; lariciresinol; secoisolariciresinol; matairesnol	Glycosylation products	Chen et al., 2021b
	UGT71B5b	MW051595			

-; not available

Phenylalanine ammonia-lyase (PAL) is the first key enzyme in the phenylalanine-derived pathway. Ma et al. (2016) and Lu et al. (2006a) isolated two PAL genes from *I. indigotica*, *IiPAL1* and *IiPAL2*; both of the recombinant proteins catalyze the conversion of l-phenylalanine to *trans*-cinnamic acid. A correlation analysis suggested that *IiPAL1* is more closely associated with the biosynthesis of secondary metabolites.

Cinnamate 4-hydroxylase (C4H) catalyzes the *para* position hydroxylation of cinnamic acid into 4*-*coumaric acid. Hu et al. (2015) cloned *IiC4H* from *I. indigotica*. Its full-length sequence is 1647 bp with an open reading frame (ORF) of 1530 bp that encodes a protein of 509 amino acids (aa). Ultraviolet-B (UV-B), methyl jasmonate (MeJA), abscisic acid (ABA), and gibberellic acid (GA_3_) treatments upregulate its transcription to varying degrees (Hu, 2010; Hu et al., 2015).

In the main pathway that generates 4-coumaroyl CoA, 4-coumarate CoA ligase (4CL) is the final enzyme. Three *Ii4CL* genes were cloned and characterized. Recombinant *Ii*4CL2 can use 4-coumaroyl acid, caffeic acid, ferulic acid, and sinapic acid as substrates, whereas *Ii*4CL3 uses only caffeic acid and ferulic acid, and *Ii*4CL1 does not use any of these four compounds as a substrate. RNA interference (RNAi) of *Ii4CL2* in hairy-root lines of *I. indigotica* showed induced accumulation of lariciresinol (maximum of 2.5-fold) relative to that in wild-type lines. In contrast, *Ii4CL3* RNAi in hairy-root lines resulted in a lower accumulation of lariciresinol (minimum of 0.4-fold) relative to wild-type lines. Therefore, *Ii4CL3* plays a significant role in synthesis of lariciresinol (Di et al., 2012; Zhang et al., 2016a).

Coumaric acid 3-hydroxylase (C3H) catalyzes the 3-position hydroxylation of 4-coumaroyl CoA into caffeoyl CoA. Xuan (2012) isolated *IiC3H* from *I. indigotica*. Its full-length sequence is 1830 bp with an ORF of 1527 bp that encodes a 509-aa protein. Its transcription is induced by UV and MeJA treatment. Hairy-root lines that overexpress *IiC3H* show a four- and nine-fold increase in lariciresinol and sinapyl alcohol, respectively, relative to that in wild-type lines (Chen et al., 2015; Xuan, 2012).

Caffeoyl CoA 3-*O*-methyltransferase (CCoAOMT) catalyzes the conversion of deoxymethyl of caffeoyl CoA into feruloyl CoA. Li et al. (2021) cloned *IiCCoAOMT* from *I. indigotica*; it has a full-length sequence of 1098 bp with an ORF of 774 bp that encodes a 257-aa protein. Transcription of this gene is induced by low temperature and NaCl, MeJA, salicylic acid (SA), ABA, and GA_3_ treatments (Li et al., 2021; Lu, 2006).

Cinnamoyl-CoA reductase (CCR) catalyzes the nicotinamide adenine dinucleotide phosphate (NADPH)-dependent reduction of cinnamoyl CoA esters to their corresponding cinnamaldehydes, such as coniferaldehyde and 4-coumarldehyde. Hu et al. (2011) cloned *IiCCR*, which has a full-length sequence of 1368 bp and an ORF of 1026 bp that encodes a 341-aa protein. Treatment with MeJA, GA_3_, and UV-B induced *IiCCR* expression with the highest level about 1.5-fold higher than that of the wild type. In contrast, ABA had a repressive effect on *IiCCR* expression, with a minimum level about 5-fold lower than the wild type.

Cinnamyl alcohol dehydrogenase (CAD) catalyzes the conversion of cinnamyl aldehydes into their corresponding alcohols, such as *p*-coumaryl alcohol, coniferyl alcohol, and sinapyl alcohol. A single *IiCAD* gene has been cloned; it has a full-length sequence of 1042 bp and an ORF of 1083 bp that encodes a 360-aa protein. Both UV-B and MeJA induce *Ii*CAD expression to varying degrees (Hu, 2010).

Hydroxycinnamoyl-CoA shikimate/quinate hydroxycinnamoyl transferase (HCT) catalyzes two steps in the phenylpropanoid pathway, from *p*-coumaroyl CoA to *p*-coumaroyl shikimate and from caffeoyl shikimate to caffeoyl CoA in *Arabidopsis thaliana*. Decreasing the expression of HCT inhibits the accumulation of coniferyl alcohol (Besseau et al., 2007). Dong et al. (2015) isolated *IiHCT*, the homolog of *AtHCT*, and showed that its transcriptional *IiHCT* is induced by MeJA treatment in the hairy-root lines of *I. indigotica*.

Dirigent protein (DIR) catalyzes the coupling of two coniferyl alcohol molecules into pinoresinol. Li et al. (2014) and Chen (2018) identified two *IiDIR* genes (*IiDIR1* and *IiDIR2*) among the *DIR* family in *I. indigotica*that catalyze the synthesis of (−)pinoresinol. Further studies showed that *Ii*DIR2 promotes the accumulation of (+)secoisolariciresinol.

Pinoresinol/lariciresinol reductase (PLR) is involved in the transformation from pinoresinol to lariciresinol and from lariciresinol to secoisolariciresinol. Three *IiPLR* genes were isolated from *I. indigotica.* However, only *IiPLR1* was associated with the ability to inhibit accumulation of lariciresinol. The *IiPLR1* RNAi hairy-root lines showed decreased lariciresinol levels, whereas hairy-root lines that overexpressed this gene showed increased lariciresinol levels. Consistently, recombinant protein *Ii*PLR1 was able to catalyze the target reaction from pinoresinol to lariciresinol and even secoisolariciresinol in vitro (Xiao et al., 2015, 2021). Recently, crystal structures for *Ii*PLR1 in the apo, substrate-bound, and product-bound states were fully elucidated, and the molecular mechanism underlying its substrate specificity was well explained. Mutagenesis of *IiPLR1* successfully eliminates the second reaction that converts lariciresinol to secoisolariciresinol, leading to high accumulation of the pharmaceutically valuable compound lariciresinol in *E. coli* (997.79 mg/L) (Xiao et al., 2021).

UDP-glucuronosyltransferase (UGT) catalyzes the transfer of the glycosyl group from nucleoside diphosphate-activated sugars (UDP sugars as donors) to a diverse array of secondary metabolites (the acceptors). In *I. indigotica*, 147 UGTs have been identified and classified into 41 subfamilies. Transcriptome co-expression analysis showed that five genes that encode UGTs (*71C1*, *71C2*, *71D1*, *72E3*, and *84A4*) clustered with *DIR2* and *DIR3* (the downstream genes involved in the lignan biosynthesis pathway described above) and thus are regarded as lignan glucosyltransferase genes (Chen et al., 2013a). Recently, Chen et al. (2021b) identified two additional UGT-encoding genes (*71B5a* and *71B5b*) that are responsible for glycosylation at the 4-position of pinoresinol. In addition, 71B5a and 71B5b can both catalyze the production of all four compounds downstream of the lignan pathway in *I. indigotica* from pinoresinol to matairesinol.

The studies on biosynthetic pathways of active compounds provide potential points at which researchers can regulate the features of plants and thus obtain species with ideal accumulation of target compounds. For example, overexpression of *Ii*PLR1 and *Ii*C3H leads to a higher accumulation of lariciresinol in hairy roots of *I. indigotica* (Chen et al., 2015; Xiao et al., 2015). Some phytohormones or plant growth regulators such as SA and jasmonic acid (JA) act as conserved elicitors of plant secondary metabolism. Transcriptional factors often play essential roles in their signaling. In addition to activating or suppressing the pathway enzyme genes directly, means to accomplish these assignments are also based on using transcription factors which may influence the flows. Zhang et al. (2016) identified 16 *IibHLHs* (basic helix-loop-helix) out of the known 78 members of this family in *I. indigotica* that have a significant positive response to MeJA, and seven of them were highly homologous to known MYCs. A member of APETALA2/ethylene response factor (AP2/ERF), encoded by *Ii049*, was identified to be a positive regulator for accumulation of SA, and thus also for lignan content, including lariciresinol, pinoresinol, and secoisolariciresinol, and even for lignins in *I. indigotica* (Ma et al., 2017a). The transcription factor *Ii*WRKY34 was found not only to regulate lignans but also to have a positive role in root development and stress tolerances (Xiao et al., 2020) (Table 2).

**Table 2:** Regulatory genes used for genetic improvement of *I. indigotica*

Name	Gene	Gene ID	Function/Pathway	Reference
Calcium-dependent protein kinases	*CDPK1*	DQ098651	Stress-responsive pathway and hormone signaling	Lu et al., 2006b
	*CDPK2*	DQ458915		Pan et al., 2008
FRUITFUL	*FUL*	LC321987	Multiple reproductive growth stages, including floral transition, meristem determinacy, floral organ differentiation and fruit ripening	Ma et al., 2017a
SHATTERPROOF2	*SHP2*	–	Development of reproductive organs	Lu et al., 2020
SEPALLATA	*SEP1*	LC472304, LC472305		Ma et al., 2019
	*SEP4*	–		Pu et al., 2020
Flowering locus C	*FLC*	–	Flowering related protein	Wang et al., 2018
Later embryogenesis abundant proteins	*LEA*	AY866484	–	Lu et al., 2011
GRAS	*GRAS*	–	Plant growth and development	Zhang et al., 2016c
Stomatal density and distribution1	*SDD1*	DQ407741	Regulation of stomatal differentiation and pattern formation	Xiao et al., 2010
AP2/ERF Transcription Factor	*Ii049*	–	Lignan and lignin biosynthesis pathways	Ma et al., 2017
	*IiERF008*	–	*Ii*DIR related factor	Chen, 2018
WRKY Transcription Factor	*WRKY34*	MN480620	Lignan biosynthesis pathway, stress-responsive pathway and growth of root	Xiao et al., 2020
Insecticidal proteins	*Bt* and *Pta*	–	Insect-resistant proteins	Xiao et al., 2012

-; not available

### Biosynthesis and regulation of indole alkaloids

Tryptanthrin, indirubin, and indigo are the main active indole alkaloids in *I. indigotica*. Indigo and indirubin are known as products formed after fermentation of the aerial parts of this plant, whereas indican is their precursor and is stored in vacuoles and released after cell death (Chen et al., 2013a; Marcinek et al., 2000; Minami et al., 2000).

**Fig. 3:**
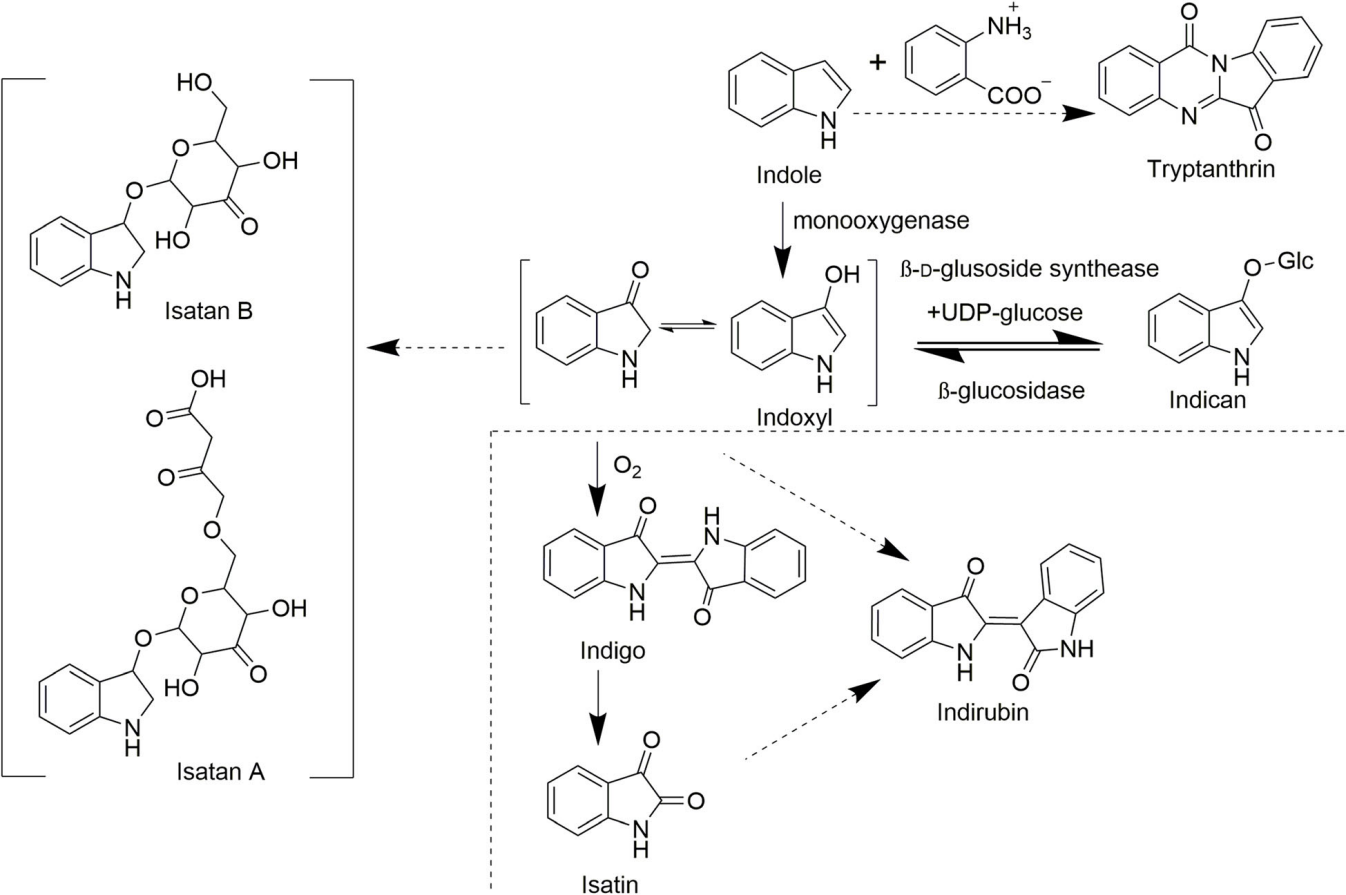
Biosynthetic pathway of indole alkaloids in *I. indigotica.* Indoxyl can be biosynthesized from indole or from the degradation of indican; the oxidation or glycosylation reaction make it stable. Indigo, indirubin, and isatin are produced outside of the cells. The figure is adapted from Maier et al. (1990) and Xia and Zenk (1992)

The synthesis of indigo, because of its widespread use as a blue dye, has aroused great interest. It is generally believed that indoxyl has an important role in addition to indican in the indigo biosynthetic pathway in *I. indigotica*. Indoxyl can be synthesized by two paths in the cell. Indoxyl can be biosynthesized from indole, an intermediate compound of the tryptophan pathway (Maier et al., 1990), and the starting point of the process (Xia and Zenk, 1992), in a step catalyzed by a monooxygenase (Fig. 3). Cytochrome P450s (CYPs) and flavin-containing monooxygenases (FMOs) are two major monooxygenase families in plants, and researchers have reported many enzymes from the two families that catalyze this step. For instance, recombinant human CYP2A6 expressed in *E. coli* can convert indole into indoxyl with NADPH cytochrome P450 reductase (NPR) in vitro (Gillam et al., 1999); this pathway has also been rebuilt successfully in a tobacco cell suspension system (Warzecha et al., 2007). Similarly, CYP102A from *Streptomyces cattleya* when expressed in *E. coli* also produces indigo (Kim et al., 2017). Additionally, *E. coli* expressing bacterial FMOs from *Methylophaga* sp., *Sphingomonas wittichi* (Singh et al., 2010), *Corynebacterium glutamicum* (Ameria et al., 2015), and *Nitrincola lacisaponesis* (Loncar et al., 2019) can produce indigo.

Alternatively, indoxyl can be synthesized from the degradation of indican as catalyzed by β-glucosidase (BGL) in *Polygonum tinctorium* (Minami et al., 1996). Indoxyl is, however, unstable and needs to be converted into indigo in the presence of oxygen or transferred to the next enzyme. The *P. tinctorium* indoxyl β-glucoside synthase (IGS) most likely carries out this step while using UDP-glucose to recover indican. The identification of protein-protein interactions between *Pt*FMO and *Pt*IGS led to this hypothesis (Inoue et al., 2021).

There are many other kinds of indole alkaloid derivatives. For example, isatans are indoxyl derivatives that differ in the moiety linked to the hydroxyl group of indoxyl-like indican. Isatan A [indoxyl-3-*O*-(6′-*O*-malonyl-β-d-ribohexo-3-ulopyranoside)] and isatan B (indoxyl-3-*O*-β-d-ribohexo-3-ulopyranoside) are two major products. The accumulation of isatan A and the ratio of indican to isatan B are critical differences between *I. indigotica* and *I. tinctoria* (Gilbert et al., 2004).

Isatin (indole-1*H*-2,3-dione) is obtained from *Isatis* plants after the oxidation of indigo is complete (Chauhan et al., 2020). Indirubin may be produced as a byproduct of isatin production. Isatin is also an endogenous component of mammalian tissues and body fluids, where it has a variety of activities such as anti-viral, anti-corrosive, and transthyretin fibrillogenesis inhibitory activity (Abbas et al., 2013; Gonzalez et al., 2009; Zhang et al., 2014). The metabolic route by which isatin is produced is not clear, although it may result from the synthesis pathway of tryptophan to indole in bacteria followed by oxidation in the liver in humans (Chauhan et al., 2020).

Unfortunately, few of the homologous genes of enzymes that participate in the indigo biosynthesis pathway have been reported for *I. indigotica*. The definitive synthesis reaction in this species needs additional investigations.

## Biotechnological methods for the improvement of *I. indigotica*

Qiao et al. (1989) obtained autotetraploid *I. indigotica* (2n = 28) using colchicine treatment after a 5-year selection. This tetraploid has significantly better yield and enhanced resistance to stress relative to its diploid progenitor (2n = 14).

Researchers used the *A. thaliana* whole-genome Affymetrix gene chip (ATH1) to survey the variation in gene expression between tetraploid and diploid *I. indigotica*. There was a coordinated induction and suppression of 715 and 251 ploidy-responsive genes, respectively, in tetraploid *I. indigotica* that are involved in various developmental, signal transduction, transcriptional regulation, and metabolic pathways (Lu et al., 2006a). Several of these genes, including the transcription factor *IiWRKY34* (Xiao et al., 2020), the stomatal developmental gene *IiSDD1* (Xiao et al., 2010), signal transduction genes *IiCPK1* and *IiCPK2* (Lu et al., 2006b; Pan et al., 2008), and the lignan biosynthetic pathway gene *IiPAL*, have been characterized to explore their contribution to the favorable physiological consequences after polyploidization (Lu et al., 2006c). In particular, *IiWRKY34* has large pleiotropic effects on an array of traits, including yield, lignan biosynthesis, and stress tolerance, which are inferred to have a substantial contribution to the high level of polyploidy vigor of *I. indigotica* (Xiao et al., 2020) (Table 2). These results may lead to gene-based, molecular marker-assisted selection and transformation for the improvement of *I. indigotica* as an alternative to individually manipulating the component traits using multiple genes with small effects.

Transcriptomic analysis was also used to explore the gene expression changes between tetraploid and diploid *I. indigotica*. The differentially expressed genes were mainly involved in cell growth, cell wall organization, secondary metabolite biosynthesis, stress response, and photosynthetic pathways (Xiao et al., 2020). Further studies are required to complete the characterization of the mechanisms of autotetraploidy vigor of *I. indigotica*, which will be helpful for identifying potential targets for genetic improvement.

Some phenotypic features are specific to *I. indigotica* compared with other plants in the Cruciferae family. These features may be caused by special genes related to growth and development. The fruits of *I. indigotica* are referred to as indehiscent silicles, whereas the typical fruits of the Cruciferae family are dehiscent siliques. These morphological variations suggest that *I. indigotica* undergoes different processes with respect to floral transition and reproductive growth. Some transcription factors related to the development of reproductive organs play important roles in the floral transition process, such as FUL (FRUITFUL), SHP (SHATTERPROOF2), and SEP (SEPALLATA). The *Ii*FUL can affect the development of leaves, florescence meristems, flowers, and fruits (Ma et al., 2017b). Plants that overexpress *Ii*SHP2 bolt earlier, produce leaves that are transformed in shape, have shorter sepals, and have unencapsulated flower buds (Lu et al., 2020). Expression of *Ii*SEP1 and *Ii*SEP4 induces early flowering and reduction of flowers and floral organs in *A. thaliana*, and overexpression of *Ii*SEPs changes the structure of sepals in *I. indigotica* (Ma et al., 2019; Pu et al., 2020). Negative regulatory factor *Ii*FLC (flowering locus C) initially decreased and then increased obviously in the development periods from bolting to fruit bearing (Wang et al., 2018). Later embryogenesis abundant (*Ii*LEA) protein did not express in the normal growth conditions in *Isatis* seedings, its expression level gradually increased with salt or drought stress time, and so may be induced by environmental stress (Lu et al., 2011). Forty-one *GRAS* genes were identified from *I. indigotica*, which may have crucial roles in diverse plant growth and development (Zhang et al., 2016c).

Currently, tetraploid *I. indigotica* is widely cultivated to meet the increasing market demand. Despite the greater yield and enhanced stress resistance of these plants, insect damage has remained a substantial problem in its cultivation, affecting both the yield and quality of this medical material. Some attempts have been made to enhance the insect resistance of *I. indigotica.* Two insecticidal genes (Bt and Pta) were simultaneously introduced into tetraploid *I. indigotica*, resulting in increased protection against both moths and aphids. This was the first attempt to engineer pest control in medicinal plants and offers an efficient molecular breeding strategy for incorporating insect tolerance into such plants (Xiao et al., 2012).

## Conclusion and perspectives

As a well-known indigo-producing and medicinal plant, *I. indigotica* has been intensively studied over the past 30 years. Great progress has been made in understanding the biosynthesis and metabolic regulation of bioactive compounds such as lignans, indole alkaloids, and their corresponding derivatives. Many key enzymes and transcription factors have been identified and shown to be efficient regulators for the accumulation of lignan and its glycosylated products. It is noteworthy that the biosynthesis pathway of indole alkaloids, including indigo, and the underlying regulatory mechanisms in *I. indigotica* are not yet fully understood. Although a considerable number of genes have been proposed to be involved in indole alkaloid biosynthesis based on the genome assembly of *I. indigotica*, their function remains to be fully explored. The construction of a “gene-metabolite network” using the available information may help to elucidate this biosynthetic pathway and thus facilitate the process of rationally designing strategies for further improvement of indole alkaloid production.

There are several issues that still need to be addressed;

1. The *I. indigotica* hairy-root culture system has been widely used in gene functional analyses and metabolic engineering. This system may not, however, be suitable for the characterization of all genes, especially those that function in the aerial parts of *I. indigotica* plants, are involved in plant development, or respond to environmental changes. As an alternative to culture systems, a genetic transformation system has been established for *I. indigotica* plants. However, the system is not very stable and requires further optimization.
2. A large number of bioactive compounds are produced by *I. indigotica*. The cellular localization and transport and the subcellular compartmentalization and trafficking of these compounds are largely unknown. Meanwhile, metabolic flux results in dynamic changes during the life cycle of all plants. There must be specific mechanisms to control such temporal and spatial profiles. Therefore, exploring the transcriptional regulation mechanism and constructing regulatory networks will be helpful for identifying the specific transcription factors that control specific metabolites.
3. The large-scale production of bioactive compounds by synthetic biology has emerged as an attractive alternative to extracts from *I. indigotica*. It has been successfully used in the production of bioactive compounds such as lariciresinol. The application of synthetic biology approaches to other bioactive compounds including a series of lignan glycosylated products and indole derivatives is a likely direction for future research.

With the information obtained from sequencing the whole genome of *I. indigotica*, more functional genes, gene families, and transcriptional regulators will be identified, which can be used to improve the quality of *I. indigotica* as an important material for traditional Chinese medicine, to facilitate the production of bioactive compounds through synthetic biology, and to support the research of other medicinally important plants.

## Data Availability

Not applicable.

## Abbreviations

ABA: abscisic acid
AP2/ERF: APETALA2/ethylene response factor
BCE: Before Common Era
bHLHs: basic helix-loop-helix
CAD: cinnamyl alcohol dehydrogenase
CCoAOMT: caffeoyl CoA 3-O-methyltransferase
CCR: cinnamoyl-CoA reductase
CDPK: calcium-dependent protein kinase
CE: Common Era
CoA: Coenzyme A
CYPs: cytochrome P450s
C3H: coumaric acid 3-hydroxylase
C4H: cinnamate 4-hydroxylase
DIR: dirigent protein
FLC: flowering locus C
FMOs: flavin-containing monooxygenases
FUL: FRUITFUL
GA_3_: gibberellic acid
HCT: hydroxycinnamoyl-CoA shikimate/quinate hydroxycinnamoyl transferase
JA: jasmonic acid
MeJA: methyl jasmonate
NPR: NADPH cytochrome P450 reductase
ORF: open reading frame
PAL: phenylalanine ammonia-lyase
PLR: pinoresinol/lariciresinol reductase
SA: salicylic acid
SEP: SEPALLATA
SHP: SHATTERPROOF2
SDD1: stomatal density and distribution1
UGT: UDP-glucuronosyltransferase
UV-B: ultraviolet-B
4CL: 4-coumarate CoA ligase
